# 2023 Acknowledgment of *mSphere Ad Hoc* Reviewers

**DOI:** 10.1128/msphere.00690-23

**Published:** 2023-12-20

**Authors:** Michael J. Imperiale

**Affiliations:** 1 University of Michigan Medical School, Ann Arbor, Michigan, USA

## EDITORIAL

As the end of 2023 approaches, I embark on my annual task of looking back at the year at *mSphere*. I am very pleased to report that our number of submissions has begun to increase again after a bit of a slump in 2022. I have been impressed both by the importance of the research we are publishing, as well as the diversity in terms of types of studies and the broad geographical location of the labs studying infectious agents.

You may have noticed that we recently started publishing a new series called Full Circle. These are minireviews from junior scientists who previously wrote about the research that excites them in our mSphere of Influence (mSI) series. We reached out to the first cohort of authors to ask them to review what has happened in their field since they wrote their mSI pieces. We think you’ll find their perspectives to be fresh and unique. This initiative will continue on a rolling basis.

Finally, as always, I want to acknowledge the many excellent reviewers who have taken the time to assist us in the peer review process. It is no secret to us editors in chief of the ASM journals, or to editors and publishers more broadly, that scientists are being asked to contribute more and more volunteer service to the field by reviewing manuscripts. This sometimes translates into small but palpable delays in getting manuscripts reviewed, because editors must make more requests to potential reviewers than in the past. The ASM Journals Committee has discussed this extensively, and there are no easy answers to this situation. All I can say is that if you have reviewed for *mSphere*, you have my thanks and the appreciation of our senior editors, editors, and, of course, the authors whose manuscripts benefit as a result of the peer review process. The individuals who served as *mSphere* reviewers during 2023 are listed here.

Sarah Adamo

Amos Adler

Muhammad Afzal

Christina Ann Ahlstrom

Waqar M. Ahmed

Kait F. Al

Ali Al-Ahmad

Richard A. Alm

Samuel Alvarez-Arguedas

Carmen Amaro

Cheryl Ames

Kingsley Kwaku Amoako

Matthew Zack Anderson

Andrey P. Anisimov

Alberto Antonelli

Gabriele Arcari

Catherine R. Armbruster

Ina Attrée

Jennifer M. Auchtung

Leonardo da Silva Augusto

Herwig Bachmann

Simon Baker

Connor Gavin George Bamford

James D. Bangs

Ravi Pratap Barnwal

Javad Barouei

Antonio Barragan

Gerald Barry

Christine Marie Bassis

Victoria K. Baxter

Josh R. Beck

Michael G. Becker

Emma Bell

Vivian Bellofatto

Jose A. Bengoechea

Gordon M. Bennett

Matthew Berens

Laura M. Bergner

Douglas A. Bernstein

Brittni Bertolet

Purnima Bhanot

Blake Billmyre

Raphael Bisinotto

Patricia Pringle Bloom

Justin A. Boddey

Lydia M. Bogomolnaya

Paul L. Bollyky

Raquel Regina Bonelli

Adrianus C. Boon

Batbileg Bor

Katherine A. Borkovich

Dustin Bosch

Cyrille Y. Botté

Philippe Bouloc

Travis Bourret

Jeff M. Boyd

Ann Karen Brassinga

Shaun R. Brinsmade

Kendall Bryant

Bryan D. Bryson

Zackery Bulman

Barbara Anne Burleigh

Laura S. Burrack

Jeremy P. Burton

Juan Calvet Seral

Miguel Camacho

Stéphane Canaan

Michael G. Caparon

Eric Caragata

George Carnell

Molly Carpenter

Ronan K. Carroll

Elizabeth Di Russo Case

Fred Cassels

Eric Cassmann

Mariana Castanheira

Stephanie Caty

Anirban Chakraborty

Emily Chase

Chen Chen

Liang Chen

Xinhua Chen

Yang Chen

Tse-Yu Chen

Loo Wee Chia

Alexandra Chiaverini

Michaelle Chojnacki

Rebecca C. Christofferson

Sarah Clark

Christine Clayton

Tom Coenye

Francesc Coll

Brendan Cormack

Pierre Cornelis

Cynthia Nau Cornelissen

Peggy A. Cotter

Laura M. Cox

Michael M. Cox

Hannah M. Creager

Bo Cui

Jie Cui

Paul J. Cullen

Leonardo Curatti

Kurt Martin Dahlstrom

France Daigle

Promi Das

Bryan W. Davies

Scott C. Dawson

Charles R. Dean

George S. Deepe

Paul De Figueiredo

Maxime Deforet

John P. Dekker

Jeseth Delgado Vela

Jill A. Dembowski

Sally Demirdjian

Hongyu Deng

Celso José Bruno De Oliveira

Nicolas De Prost

Lalitagauri Deshpande

Berthony Deslouches

Floyd E. Dewhirst

Stephanie Dewitte-Orr

Robert P. Dickson

Sara Dirienzi

Julianne T. Djordjevic

Monika Dolejska

Anna Dongari-Bagtzoglou

Sheila Donnelly

Zhicheng Dou

Timothy B. Doyle

Rebecca Drummond

Jingjie Du

Jinju Duan

Lidiya Dubytska

Adrien Ducret

Seána Duggan

Sherry A. Dunbar

Brianna Duncan-Lowey

Jonas Dutra Albarnaz

Uta Effmert

Waldir P. Elias

Jeremy R. Ellermeier

Marie A. Elliot

Iuliana V. Ene

J. Pamela Engelberts

Cecilie Ersdal

Emilie Esnault

Eduardo Espeso

Mariana Bossi Esteves

Benjamin Ezraty

Patrizia Falabella

Hongjie Fan

Rhys A. Farrer

Joseph Fauver

Ian M. Feavers

Kenneth A. Fields

Maria F. Fillat

Scott G. Filler

Reinhard Fischer

Ross Fitzgerald

James Michael Fleckenstein

Mike Flint

Andrea Fogaça

Mattias N. E. Forsell

Elizabeth M. Fozo

Michael T. France

Maria E. Francia

Dara W. Frank

Carl Johan Franzén

Friedrich Frischknecht

Kevin Fuller

Sarah L. Gaffen

Raj Gaji

Emily Gallichotte

João Alves Gama

Rodolfo Garcia-Contreras

Courtney Gardner

Amy Shirley Gargis

Emily Garner

Alberto Garre

Uma Shankar Gautam

Christian Gauthier

Michael Gebhardt

Otto Geiger

Christiane Gerke

Jimut Kanti Ghosh

Partho Ghosh

Sebastian Giese

Rosario Gil

Sabine Gilch

Paul R. Gilson

Virginia E. Glazier

Nathan Glueck

William E. Goldman

Stephen Goldstein

Vijay Gondil

Ana S. Gonzalez-Reiche

Ria Goswami

Revathi Govind

Keith D. Green

Trudy H. Grossman

Rajesh Gupta

Sarah-Jane Haig

Anders P. Hakansson

James P. J. Hall

Rebecca A. Hall

Michael R. Hamblin

Mehrad Hamidian

Neal D. Hammer

Katherine R. Hargreaves

Lucas B. Harrison

Erica M. Hartmann

Nur A. Hasan

Masanori Hatakeyama

Terry C. Hazen

Bin He

Mary Healy

Joseph Heitman

Dag Hessen

Julian F. Hillyer

Kevin Loren Hockett

Anders Hofer

Jeffrey Holloman

Michael J. Holmes

Matthias Horn

Galadriel Astra Hovel-Miner

Kate Joanne Howell

Ke Hu

Victor C. Huber

Dana E. Hunt

Kevin Hybiske

Carolyn Ibberson

Ashraf Ibrahim

Cheryl Ingram-Smith

Bungonsiri Intra

Ralph R. Isberg

William T. Jackson

Timothy James

Byeonghwa Jeon

Min Jin

Jörgen Johansson

Jeremiah G. Johnson

Reed F. Johnson

Won Hee Jung

David Kadosh

Jessica K. Kajfasz

René Kallies

Jana Kamanova

Donghoon Kang

John Y. Kao

David Karig

Maria Karlsson

Justin R. Kaspar

Mark D. Kellogg

Alyson A. Kelvin

Chelsea Kettering

Jessica Kevill

Asad U. Khan

Nicolas Kieffer

Min-Ju Kim

Clare L. Kinnear

Tomoe Kitao

Michele M. Klingbeil

Anna Kloc

Larry K. Kociolek

Carolina M. Koeller

Michael H. Kogut

Tse Hsien Koh

Julia Ruth Köhler

Xiangfeng Kong

James B. Konopka

Ákos T. Kovács

Jens Kreth

James W. Kronstad

Anand Kumar

Purnima S. Kumar

Kun Kun

Hiroyuki Kunishima

Gyanu Lamichhane

Ryan A. Langlois

Andrew Lee

Soo Chan Lee

Vincent T. Lee

Mckenzie K. Lehman

Valerie M. Le Sage

Julian L. Leibowitz

Jorge H. Leitão

Manuel L. Lemos

Jay T. Lennon

Gina R. Lewin

Diyan Li

Jianrong Li

Xian-Zhi Li

Xinlou Li

Yongxin Li

Yongheng Liang

Joanito Liberti

Tami Lieberman

Krista Liguori

Shen Jean Lim

Bruno P. Lima

Senjie Lin

Jodi A. Lindsay

Anastasia P. Litvintseva

Cong Liu

Jia Liu

Qin Liu

Yi-Fan Liu

Cheryl A. Lobo

Allison Lopatkin

Christopher A. Lopez

Lorena López-Cerero

Alexander Lorenz

Tillmann Lueders

Anja Lührmann

Valeria Lulla

Christine Luttermann

Julia Lynch

Zhe Ma

Donna M. MacCallum

Milton Maciel

Roderick I. Mackie

N. James MacLachlan

Paul Magwene

Johan Malmström

Francesca Mancini

Costas D. Maranas

Lucie Maranda

Nicholas O. Markham

Richard Markham

Mary E. Marquart

Eric Martens

Miguel A. Martín-Acebes

Juan Jose Martinez

Luis R. Martinez

Bruno Martorelli Di Genova

Preston Marx

Jyl S. Matson

Cyrille Matthieu

Anthony T. Maurelli

Kyle M. J. Mayers

Jere W. McBride

Mark J. McBride

Shonna M. McBride

John K. McCormick

Bruce McDonald

Patrick McGann

Martin J. McGavin

Bridget B. McGivern

Elizabeth A. McGraw

Debbie McKenzie

Anna L. McLoon

Ane Hackbart de Medeiros

Supriya D. Mehta

Ran Mei

Lulu Meng

Tod J. Merkel

D. Scott Merrell

Robert C. Mettelman

Craig Meyers

Firas S. Midani

Eric L. Miller

Eliane Milohanic

Pedro Miramón

Maria F. Mojica

Michelle Momany

Hector M. Mora-Montes

Cristiano Gallina Moreira

Tiago Facury Moreira

James C. Morris

John Morrison

Thomas E. Morrison

Paul Moss

Caroline Mota

W. Scott Moye-Rowley

Sergio Munoz Gomez

Carol A. Munro

Nontobeko Eunice Mvubu

Michael Angelou L Nada

Moon H. Nahm

Hiroaki Naka

Jacob T. Nearing

David E. Nelson

Ashley Nelson

Jeniel E. Nett

Hayley Joy Newton

Caroline L. Ng

Jeppe Nielsen

Marisa C. Nielsen

Leonardo Nimrichter

Corey Nislow

Diego Nobrega

Carolina Silva Nodari

Douglas E. Norris

Eric Nuermberger

Austin S. Nuxoll

David O’Callaghan

Allyson F. O'Donnell

Sarah O'Flaherty

Kenta Okamoto

Michael Olson

Daisuke Ono

Joshua Osowicki

Ana Beatriz Furlanetto Pacheco

Samantha G. Palace

Jana Palkovičová

Kelli L. Palmer

Sneh Lata Panwar

Daniel Paredes-Sabja

Alexander R. Paredez

Geun Woo Park

Hee-Soo Park

Dane Parker

Srivatsan Parthasarathy

Fabienne Paumet

Talima Pearson

Miguel A. Penalva

Yousong Peng

J. Christian Pérez

Lena Pernas

Brian M. Peters

Hadrien Peyret

Niels Pfenningwerth

Marcelo Pillonetto

Daniel Pletzer

Laurent Poirel

Saskia Popescu

Megan Povelones

Guruswamy Prabhakaran

Chinnamani Prasanna Kumar

Sladjana Prisic

Michael J. Pucci

Charles Puerner

Joanna A. Pulit-Penaloza

Alexandra E. Purdy

Georgiana E. Purdy

John G. Purdy

Dohun Pyeon

Wenjie Qiao

Yafeng Qiu

Kumaran S. Ramamurthi

Mika Rämet

Maria Soledad Ramirez

Reeta Prusty Rao

Xiancai Rao

Viduthalai Rasheedkhan Regina

Carol Rauch

Laurie K. Read

Michelle Lynne Reniere

Sandra Reuter

Johanna Rhodes

Jason M. Ridlon

Bert K. Rima

Amariliz Rivera

Cathy Robinson

Mélanie Roch

Daniel D. Rockey

Eduardo Rodríguez-Román

Elizabeth E. Rogers

P. David Rogers

Robin Rebecca Rohwer

Sandra Romero-Steiner

Emily E. Rosowski

Cyprian C. Rossetto

Francois Rousset

Laura N. Rusche

Juliana Sá

Marat R. Sadykov

David Safronetz

Wilmara Salgado

Lamba Sangare

Priscilla A. San Juan

Felipe H. Santiago-Tirado

Yuya Sato

Karen M. Scanlon

Daniel H. Scharf

Jeffrey W. Schertzer

Patrick D. Schloss

Jonathan E. Schmitz

Michael Schotsaert

Alexander Schouten

Stefan Schwarz

Uli Schwarz-Linek

Jessica A. Scoffield

Anna Maria Seekatz

Ryan F. Seipke

Adnane Sellam

Julio Sempere

William M. Shafer

Stephanie R. Shames

Brian D. Shaw

Aimee Shen

Weishou Shen

Sunny Shin

Dmitry Shvarev

Upinder Singh

Ritam Sinha

Ciaran Skerry

Joan L. Slonczewski

Theo H. M. Smits

Evgeni Sokurenko

Isabel Sola

Nicolas Soler

Lynn Soong

Joseph A. Sorg

Magnus Steigedal

Hans Steinsland

David Cole Stevens

Kyle B. Stirling

Paul Stoodley

Xingmin Sun

Yi-Cheng Sun

Dongchang Sun

Kara A. Swenson

Francois-Etienne Sylvain

Tatyana A. Sysoeva

Fikadu G. Tafesse

Makoto Takeda

Toru Takeshita

Satoshi Tamazawa

Ranjan Tamuli

Kai Sen Tan

Shumin Tan

Caroline Tapparel

Paulo José Pereira Lima Teixeira

Lesly A. Temesvari

Gerard Terradas

Kevin R. Theis

Nina V. Tikunova

Rebekah Tiller

Marcelo Tolmasky

Vincenzo Torraca

Andrej Trauner

Pablo Tsukayama

Kyoko Tsukiyama-Kohara

Burkhard Tuemmler

Stephen W. Tuffs

Sondra Turjeman

Taher Uddin

Anne-Catrin Uhlemann

Jane Usher

Robert Valeris-Chacin

Bram Van den Bergh

Brian C. VanderVen

Patrick Van Dijck

Sigrid van Grinsven

Christiaan van Ooij

Jan-Peter van Pijkeren

Norman van Rhijn

Douwe van Sinderen

Daria Van Tyne

Ashley Vaughan

Roberto Vazquez-Munoz

Roberto A. Viau

Jorge E. Vidal

Jay Vornhagen

Wilna Vosloo

Aurele Vuillemin

William G. Wade

Seth T. Walk

Mark J. Walker

Richard Walker

Nicholas A. Wallace

Pengfei Wang

Yi-Wen Wang

Yifan Wang

Yong Wang

Yue Wang

Zheng Wang

Matthew J. Wargo

Mary Weber

Jianchao Wei

Kuo-Feng Weng

Robert T. Wheeler

Jeffrey D. Whitman

Darin Wiesner

Matthias Wietz

Emma H. Wilson

Jeffrey H. Withey

Christiane Wolz

Joseph Tin Yum Wong

R. Mark Wooten

Micah Worley

Michael Worobey

Rachel A. F. Wozniak

Erik Scott Wright

Gerard D. Wright

Longhua Wu

Qinglong Wu

Dennis Wykoff

Emanuel Wyler

Guoqing Xia

Yan Xiang

Fuguo Xing

Yan Q. Xiong

Chaoyang Xue

Kyosuke Yamamoto

S. Steve Yan

Jason H. Yang

Jinkui Yang

Jun Yang

George S. Yap

Min Yap

Oded Yarden

Nicole Yates

Yinyin Ye

Yunsong Yu

Ze-Fen Yu

Muhammad Ammar Zafar

Merve S. Zeden

Michael A. Zeller

Lin Zeng

Guoquan Zhang

Rui Zhang

Weiwei Zhang

Kelei Zhao

Beiwen Zheng

Jie Zheng

Wei Zheng

Bin Zhou

Baoli Zhu

Chao Zhuo

Franz Georg Zingl

